# Preeclampsia, Fetal Growth Restriction, and 24-Month Neurodevelopment in Very Preterm Infants

**DOI:** 10.1001/jamanetworkopen.2024.20382

**Published:** 2024-07-05

**Authors:** Jennifer Check, Coral Shuster, Julie Hofheimer, Marie Camerota, Lynne M. Dansereau, Lynne M. Smith, Brian S. Carter, Sheri A. DellaGrotta, Jennifer Helderman, Howard Kilbride, Cynthia M. Loncar, Elisabeth McGowan, Charles R. Neal, T. Michael O’Shea, Steven L. Pastyrnak, Stephen J. Sheinkopf, Barry M. Lester

**Affiliations:** 1 Department of Pediatrics, Division of Neonatology, Atrium Health Wake Forest Baptist, Winston-Salem, North Carolina; 2 Department of Pediatrics, Women and Infants Hospital, Providence, Rhode Island; 3 Department of Pediatrics, University of North Carolina and Chapel Hill School of Medicine, Chapel Hill; 4 Department of Psychiatry and Human Behavior, Alpert Medical School of Brown University, Providence, Rhode Island; 5 Department of Pediatrics, Harbor-UCLA Medical Center, Torrance, California; 6 Department of Pediatrics-Neonatology, Children’s Mercy Hospital, Kansas City, Missouri; 7 Department of Pediatrics, Alpert Medical School of Brown University, Providence, Rhode Island; 8 Department of Pediatrics, University of Hawaii John A. Burns School of Medicine, Honolulu; 9 Department of Pediatrics, Spectrum Health-Helen DeVos Hospital, Grand Rapids, Michigan; 10 Thompson Center for Autism & Neurodevelopment, University of Missouri, Columbia

## Abstract

**Importance:**

Preeclampsia has direct influences on a developing fetus and may impact postnatal health, and fetal growth restriction (FGR) is often seen co-occurring with preeclampsia. The development of children born very preterm after preeclampsia diagnosis with and without FGR is not well characterized.

**Objective:**

To examine the associations of preeclampsia and FGR with developmental and/or behavioral outcomes in a cohort of very preterm infants.

**Design, Setting, and Participants:**

In this cohort study, infants in the prospective Neonatal Neurobehavior and Outcomes in Very Preterm Infants study were enrolled between April 2014 and June 2016 from 9 US university-affiliated neonatal intensive care units (NICUs). Eligible infants were born before 30 weeks’ gestation. Infants were excluded for any major congenital anomalies and for maternal age younger than 18 years or cognitive impairment impacting the ability to provide informed consent. Data analysis was performed from November 2023 to January 2024.

**Exposure:**

Maternal preeclampsia and FGR in very preterm infants.

**Main Outcomes and Measures:**

The Bayley-III cognition, motor, and language scores less than 85 (−1 SD) indicated developmental delay. Child Behavior Checklist/Preschool 1.5-5 T-scores greater than or equal to 64 for internalizing, externalizing, or total problems indicated clinical importance.

**Results:**

Of 704 infants enrolled, 529 (mean [SD] gestational age, 27.0 [1.9] weeks; 287 male [54.3%]) were studied at 24-month follow-up. A total of 94 infants’ mothers had preeclampsia (23.2%), and 46 infants (8.7%) had FGR. In adjusted models, preeclampsia was not associated with Bayley-III (cognitive, *B* = 3.43 [95% CI, −0.19 to 6.66]; language, *B* = 3.92 [95% CI, 0.44 to 7.39]; motor, *B* = 1.86 [95% CI, −1.74 to 5.47]) or Child Behavior Checklist/Preschool 1.5-5 (internalizing, *B* = −0.08 [95% CI, −2.58 to 2.73]; externalizing, *B* = 0.69 [95% CI, −1.76 to 3.15]; total, *B* = 0.21 [95% CI, −2.48 to 2.91]) outcomes. FGR was associated with significantly lower Bayley-III scores (cognitive, *B* = −8.61 [95% CI, −13.33 to −3.89]; language, *B* = −8.29 [95% CI, −12.95 to −3.63]; motor, *B* = −7.60 [95% CI, −12.40 to −2.66]), regardless of preeclampsia status.

**Conclusions and Relevance:**

In this cohort study of preterm infants, preeclampsia was not associated with developmental and/or behavioral outcomes, but infants with FGR may be prone to developmental delays. These findings suggest future areas of research for understanding the roles of preeclampsia and FGR separately and together in early child development for preterm infants.

## Key Points

**Question:**

Are preeclampsia and fetal growth restriction associated with developmental and/or behavioral outcomes in very preterm infants?

**Findings:**

In this cohort study of 529 very preterm infants, the presence of preeclampsia was not significantly associated with neurodevelopmental or behavioral outcomes at 24 months corrected age. The presence of fetal growth restriction was associated with poor neurodevelopment, but not behavioral outcomes, at 24 months corrected age.

**Meaning:**

The findings of this study were inconclusive but suggest that infants with fetal growth restriction may be prone to developmental delays.

## Teaser

This cohort study examines the associations of preeclampsia and fetal growth restriction with developmental and behavioral outcomes in very preterm infants evaluated at age 24 months.

## Introduction

Preeclampsia is a multifactorial illness of pregnancy that affects 2% to 8% of pregnancies in the world^1,2,3^ and in severe forms can lead to placental and other organ injury. The effects of preeclampsia on the intrauterine environment, fetal growth, and neurodevelopment remain incompletely characterized. It is unclear what leads to the development of preeclampsia, but it is hypothesized that placental ischemia is caused by impaired spiral artery remodeling,^4^ as evidenced by abnormal umbilical artery findings on Doppler imaging.^5^ In a preeclampsia animal model, the offspring of rats with preeclampsia showed substantial delay in brain development with disruption in neurogenesis compared with the offspring of rats without preeclampsia.^6^ Pregnant people with preeclampsia had abnormal placental blood flow, as assessed with umbilical artery Doppler imaging,^7,8^ which can decrease oxygen and nutrient delivery to the fetus. There is evidence that preeclampsia alters functional brain connectivity in the human fetus during brain development.^9^ Furthermore, brain magnetic resonance imaging obtained at age 7 to 10 years in children born to mothers with preeclampsia demonstrate aberrations in white matter volumes and in cerebral vascularity.^10^ Collectively, these findings of adverse placental blood flow in the setting of preeclampsia, altered brain development in animal and fetal preeclampsia studies, and evidence of structural brain changes in childhood after exposure to preeclampsia lead to questions regarding outcomes in long-term neurodevelopment.

The potential impact of preeclampsia on long-term behavioral and developmental outcomes in humans is less clear, particularly in preterm infants. preeclampsia is associated with adverse neurodevelopmental outcomes in full-term and late preterm infants,^11,12,13^ although findings may be impacted by a multitude of factors, including degree of prematurity, fetal growth restriction (FGR), and socioeconomic factors.^14,15,16,17,18,19,20^ For example, preeclampsia has been associated with a nearly 3-fold higher risk of FGR,^21^ which itself has been associated with adverse cognitive outcomes in children.^22^ FGR has also been accompanied by poor fetal brain growth, as evidenced in small birth head circumference, as well as restricted early childhood head growth that may impact later cognition.^23^ Differentiating the unique and interacting associations of these risk factors (preeclampsia and FGR both separately and together) in the context of the associations between prematurity with developmental and behavioral outcomes therefore becomes challenging. Despite the inherent challenges, identifying targeted exposures that may lead to adverse outcomes for preterm infants with varied and cooccurring complications is paramount to preventing early delays with focused interventions.

The objective of this study was to examine the associations between preeclampsia and FGR, both separately and together, with early developmental and behavioral outcomes in very preterm infants born at less than 30 weeks’ gestation enrolled in a prospective multisite study. We hypothesized that infants born to mothers with preeclampsia and those with FGR would have poorer developmental and behavioral outcomes at 24 months corrected age compared with infants born to mothers without preeclampsia or FGR and that infants born to mothers with both preeclampsia and FGR would have the most severe developmental delays and/or behavioral problems.

## Methods

### Study Population

In this cohort study, participants in the multisite Neonatal Neurobehavior and Outcomes in Very Preterm Infants (NOVI) Study were enrolled between April 2014 and June 2016 from 9 US university-affiliated neonatal intensive care units (NICUs). Inclusion criteria were birth at less than 30 weeks’ gestation, parental fluency in English or Spanish, and living within 3 hours from the hospital and follow-up clinic site. Infants were excluded if they had major congenital anomalies, maternal age younger than 18 years, or maternal cognitive impairment impacting the ability to provide informed consent. Once attending neonatologists determined that infants were likely to survive to discharge, parents of eligible infants were invited to participate. Written informed consent was obtained from the mother, and the study was approved by each local institutional review board. Participants were included if they had preeclampsia information recorded and completed a follow-up visit at 24 months corrected age. This study followed the Strengthening the Reporting of Observational Studies in Epidemiology (STROBE) reporting guidelines for cohort studies.

### Measures

Maternal and neonatal information, including demographic, socioenvironmental, and medical data, were collected through standard procedures described elsewhere.^24,25^ Additional details are provided later in this article.

#### Maternal and Neonatal Characteristics

Information about the presence or absence of maternal preeclampsia was collected from the neonatal history and NICU admission medical records during medical record review. FGR (defined as birth weight less than the 10th percentile for gestational age)^26^ was based on Fenton^27^ growth curves.

#### Severe Neonatal Medical Morbidities

To account for neonatal medical morbidity, a published neonatal medical risk index^28^ was used, which accounts for (1) brain injury, including parenchymal echodensity, periventricular leukomalacia, and moderate-to-severe ventriculomegaly^29^; (2) severe retinopathy of prematurity; (3) bronchopulmonary dysplasia; and (4) culture-positive sepsis or necrotizing enterocolitis. Each medical complication present is counted as 1 point in the cumulative neonatal medical risk index.

#### Neonatal Neurobehavior

The NICU Network Neurobehavioral Scale (NNNS) is a neonatal neurobehavioral assessment measuring an infant’s active and passive tone, primitive reflexes, movement, attention to visual and auditory stimuli, social behavior, and a checklist of stress signs organized by organ systems.^30^ This standardized and well-validated assessment is a 20- to 30-minute procedure administered by certified examiners blinded to medical history and performed during the week of NICU discharge.^31^ The individual items are computed into 12 summary scores and then converted into discrete, mutually exclusive latent profiles of infants with similar patterns of neurobehavior.^32,33^ In our prior work,^25,32^ we identified 6 NNNS neurobehavioral profiles, 2 of which are considered dysregulated (because of either hypoarousal or hyperarousal). Because these dysregulated profiles have previously been shown to contribute to 24-month outcomes,^32^ we included NNNS hypoaroused and hyperaroused neurobehavior as covariates in this analysis.

### Outcome Measures at 24 Months

#### Developmental Assessment

The Bayley Scales of Infant and Toddler Development, Third Edition (Bayley-III),^34^ is a developmental assessment that has been validated among infants across varied medical and psychosocial risk spectra, including those born very preterm. Certified Bayley-III examiners who were masked to medical history administered study assessments. The Bayley-III yields composite scores for cognition, language, and motor development, and composite scores less than 85 (−1 SD) indicate clinically meaningful developmental delay.

#### Behavioral Assessment

The Child Behavior Checklist/Preschool 1.5-5 (CBCL)^35^ is a parent-report questionnaire rating their young child’s behavior, with raw scores converted to norm-referenced T scores for internalizing problems, externalizing problems, and total problems. T scores of 64 or higher indicate clinically meaningful problems.

### Statistical Analysis

Data analysis was performed from November 2023 to January 2024. We analyzed demographic and medical characteristics of infants and their mothers with and without preeclampsia, utilizing 2-sided *t* tests for continuous variables and 2-sided Pearson χ^2^ tests for categorical variables. For these demographic comparisons and all models moving forward, one variable was created to identify participants from minoritized racial and ethnicity groups, given the small cell size for many of the categories provided during data collection. Race and ethnicity were reported by the mother and are included to further describe the NOVI cohort. To test study hypotheses, we first used generalized estimating equation (GEE) models, to account for nesting of children within families (for multiple births) and study site, to examine preeclampsia status in association with developmental outcomes on the Bayley-III and CBCL (6 models). Next, we estimated GEE models to examine the independent effects of preeclampsia and FGR in association with 24-month Bayley-III and CBCL outcomes, modeled continuously, while nesting for multiple birth families and site (6 models). These 6 models were then adjusted for covariates, including infant biological sex, gestational age, hypoaroused and hyperaroused NNNS profiles,^30^ cumulative neonatal medical morbidities,^28^ caregiver partner status, low socioeconomic status (defined as Hollingshead level V, based on maternal education and occupation),^36^ chorioamnionitis, antenatal steroids, maternal prepregnancy obesity, and maternal age at childbirth while also accounting for nesting of multiple birth families and site. Finally, we estimated a set of GEE models examining the interaction between preeclampsia and FGR in association with 24-month Bayley-III and CBCL outcomes, modeled continuously. The level of statistical significance was adjusted using a Bonferroni adjustment to account for 6 GEE models being conducted in the primary analyses (adjusted α = .008). A sensitivity analysis was conducted to examine whether inclusion of medical morbidities and/or neonatal neurobehavior (2 variables potentially on the causal pathway between preeclampsia or FGR and developmental outcomes) because the covariates may have influenced the results from adjusted models. Statistical analyses were performed using SPSS statistical software version 28 (IBM).^37^

## Results

Of the 704 infants enrolled in the NOVI study, 529 participants (mean [SD] gestational age, 27.0 [1.9] weeks; 287 male [54.3%]) contributed data regarding maternal preeclampsia status and had 24-month follow-up data, warranting inclusion in the present study (Figure). Table 1 reports demographic information for the included vs excluded participants for whom maternal preeclampsia status or 24-month follow-up data were not available. Excluded mothers were less likely to be single parents, and excluded infants were more likely to have a brain injury. Ninety-four (23.2%) of the infants in the cohort were born to mothers with preeclampsia, and 46 infants (8.7%) received a diagnosis of FGR. Twenty-one infants with FGR were born to mothers with preeclampsia. Table 2 presents additional medical and demographic information about the sample. The gestational age was higher for infants born to mothers with preeclampsia vs those born to mothers without preeclampsia (mean [SD], 27.6 [1.6] weeks vs 26.9 [1.9] weeks). Infants born to mothers with preeclampsia weighed significantly less at birth compared with infants born to mothers without preeclampsia (mean [SD], 862 [239] g vs 962 [283] g). Table 3 presents additional statistical comparisons between infants born to mothers with and without preeclampsia on maternal and infant characteristics.

**Figure.**
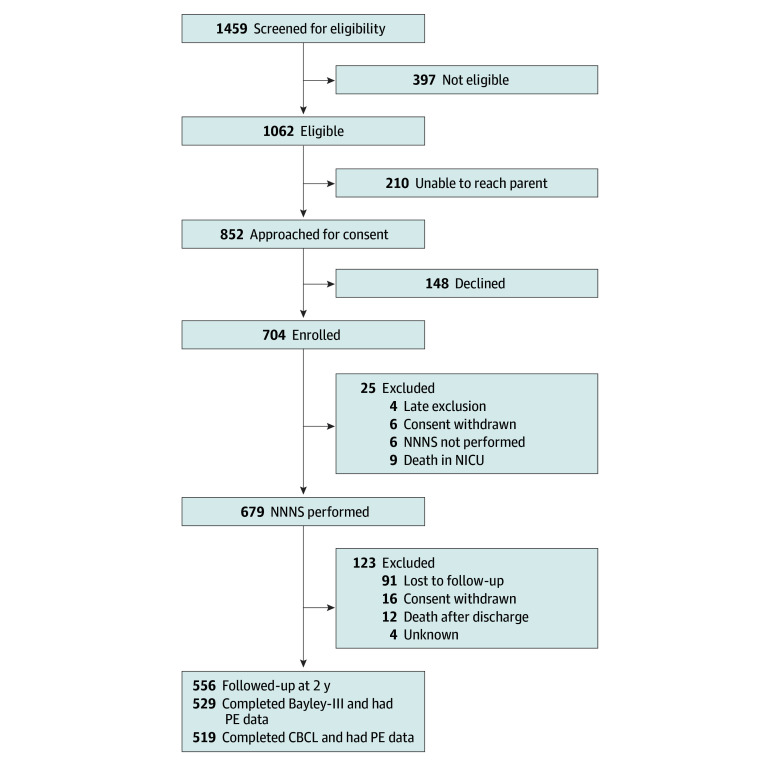
Participant Flowchart CBCL indicates Child Behavior Checklist/Preschool; NICU, neonatal intensive care unit; NNNS, NICU Network Neurobehavioral Scale; PE, preeclampsia.

**Table 1.** Demographic and Medical Characteristics of the Sample in Included vs Excluded Participants

Characteristic	Participants, No. (%)		*P* value
	Included	Excluded	
Infant characteristics			
No.	529	175	NA
Multiple gestation	144 (27.2)	40 (23.7)	.36
Fetal growth restriction	46 (8.7)	12 (7.1)	.50
Male sex	287 (54.3)	102 (60.4)	.17
Gestational age, mean (SD), wk	27.03 (1.91)	26.93 (1.94)	.55
Birth weight, mean (SD), g	943 (277)	964 (290)	.39
NNNS hypoaroused neurobehavior	32 (6.0)	15 (12.8)	.10
NNNS hyperaroused neurobehavior	116 (21.9)	43 (29.7)	.15
Severe retinopathy of prematurity	31 (5.9)	10 (5.9)	.98
Necrotizing enterocolitis and/or sepsis	96 (18.1)	32 (18.9)	.83
Bronchopulmonary dysplasia	269 (50.9)	88 (52.1)	.80
Brain injury	57 (10.8)	35 (21.0)	<.001
Maternal characteristics			
No.	405	196	NA
Maternal age at birth, mean (SD), y	28.9 (6.4)	28.7 (6.4)	.76
Minoritized racial or ethnic group	226 (55.8)	121 61.7)	.17
Single parent household	113 (27.9)	39 (20.0)	.04
Lowest socioeconomic status, Hollingshead education and occupation	41 (10.1)	18 (9.3)	.75
Education less than high school or General Educational Development	55 (13.6)	24 (12.4)	.67
Low income, public assistance, Medicaid, or uninsured	261 (64.4)	128 (65.6)	.77

Abbreviations: NA, not applicable; NNNS, NICU Network Neurobehavioral Scale.

**Table 2.** Demographic and Medical Characteristics

Characteristic	Participants, No. (%)
Infants (n = 529)	
Multiple gestation	144 (27.2)
Fetal growth restriction	46 (8.7)
Sex	
Female	241 (45.6)
Male	287 (54.3)
Race	
American Indian or Alaskan Native	1 (0.2)
Asian	25 (4.7)
Black	106 (20.0)
Native Hawaiian or Pacific Islander	2 (0.4)
White	249 (47.1)
>1	105 (19.8)
Unknown or not reported	41 (7.8)
Ethnicity	
Hispanic or Latino	119 (22.5)
Not Hispanic or Latino	410 (77.5)
Gestational age, mean (SD), wk	27.03 (1.9)
Birth weight, mean (SD), g	943.06 (277.5)
Cerebral palsy diagnosis	73 (13.8)
NNNS hypoaroused neurobehavior	32 (6.0)
NNNS hyperaroused neurobehavior	116 (21.9)
Severe retinopathy of prematurity^a^	31 (5.9)
Necrotizing enterocolitis and/or sepsis^a^	96 (18.1)
Bronchopulmonary disease^a^	269 (50.9)
Brain injury	57 (10.8)
Mothers (n = 405)	
Preeclampsia	94 (23.2)
Hypertension (chronic or pregnancy induced)	124 (30.6)
Maternal age at birth, mean (SD), y	28.9 (6.4)
Race	
American Indian or Alaskan Native	2 (0.5)
Asian	23 (5.7)
Black	91 (22.5)
Native Hawaiian or Pacific Islander	5 (1.2)
White	200 (49.4)
>1	40 (9.9)
Unknown or not reported	44 (10.9)
Ethnicity	
Hispanic or Latino	79 (19.5)
Not Hispanic or Latino	326 (80.5)
Single parent household	113 (27.9)
Lowest socioeconomic status, Hollingshead education and occupation	41 (10.1)
Education less than high school or General Educational Development	55 (13.6)
Low income, public assistance, Medicaid, or uninsured	261 (64.4)

Abbreviation: NNNS, NICU Network Neurobehavioral Scale.

^a^ These conditions are summed to determine cumulative medical morbidities on the Bassler Index.^28^

**Table 3.** Comparison of Demographic Characteristics Between Those With and Without PE

Characteristic	Participants, No. (%)		*P* value
	Mothers with PE (n = 94)	Mothers without PE (n = 308)	
Maternal characteristics			
Maternal age, mean (SD), y	29.7 (5.9)	28.7 (6.5)	.15
Maternal minoritized race or ethnicity	49 (52.1)	175 (56.8)	.42
Low income, public assistance, Medicaid	50 (53.2)	208 (67.5)	.01^a^
Education less than high school	12 (12.9)	43 (14.0)	.79
Infant characteristics			
No.	105	421	NA
Sex			
Female	55 (52.4)	186 (44.2)	.13
Male	50 (47.6)	235 (55.8)	
Birth weight, mean (SD), g	862 (239)	962 (283)	<.001^a^
Gestational age, mean (SD), wk	27.6 (1.6)	26.9 (1.9)	<.001^a^
Fetal growth restriction	21 (20.0)	25 (6.0)	<.001^a^
Severe retinopathy of prematurity^b^	5 (4.8)	26 (6.2)	.58
Bronchopulmonary disease^b^	55 (52.4)	214 (50.8)	.78
Necrotizing enterocolitis and/or sepsis^b^	17 (16.2)	79 (18.8)	.54
Brain injury^b^	10 (9.5)	47 (11.2)	.62

Abbreviations: NA, not applicable; PE, preeclampsia.

^a^ *P* < .05.

^b^ These conditions are summed to determine cumulative medical morbidities on the Bassler index.^28^

In unadjusted models examining only associations between maternal preeclampsia and 24-month corrected age developmental outcomes, there were no significant associations between preeclampsia and Bayley-III or CBCL scores (eTable 1 in Supplement 1). In unadjusted models with both preeclampsia and FGR, the presence of FGR was associated with significantly lower Bayley-III cognitive, language, and motor composite scores (eTable 2 in Supplement 1). In models adjusted for covariates (Table 4), associations between FGR and Bayley-III scores remained significant such that infants with FGR had lower cognitive (*B*** **=** **−8.61; 95% CI, −13.33 to −3.89; *P* < .001), language (*B*** **=** **−8.29; 95% CI, −12.95 to −3.63; *P* < .001), and motor (*B*** **=** **−7.60; 95% CI, −12.40 −2.66; *P* = .003) composite scores. After adjustment for covariates and Bonferroni correction, there were no significant associations between FGR and CBCL scores, or between preeclampsia and any Bayley-III (cognitive, *B* = 3.43 [95% CI, −0.19 to 6.66]; language, *B* = 3.92 [95% CI, 0.44 to 7.39]; motor, *B* = 1.86 [95% CI, −1.74 to 5.47]) or CBCL (internalizing, *B* = −0.08 [95% CI, −2.58 to 2.73]; externalizing, *B* = 0.69 [95% CI, −1.76 to 3.15]; total, *B* = 0.21 [95% CI, −2.48 to 2.91]) outcomes (Table 4). The interaction between preeclampsia and FGR on 24-month outcomes was not significant for any Bayley-III or CBCL outcomes (eTable 3 in Supplement 1).

**Table 4.** Adjusted Models Examining Associations Between PE and FGR With 24-Month Corrected Age Developmental Outcomes^a^

Measure and outcome	*B* (SE) [95% CI]	*P* value
Bayley Scales of Infant and Toddler Development, Third Edition		
Cognitive		
PE	3.43 (1.65) [−0.19 to 6.66]	.04
FGR	−8.61 (2.44) [−13.33 to −3.89]	<.001^b^
Language		
PE	3.92 (1.77) [0.44 to 7.39]	.03
FGR	−8.29 (2.38) [−12.95 to −3.63]	<.001^b^
Motor		
PE	1.86 (1.84) [−1.74 to 5.47]	.31
FGR	−7.60 (2.52) [−12.40 to −2.66]	.003^b^
Child Behavior Checklist/Preschool 1.5-5		
Internalizing		
PE	−0.08 (1.36) [−2.58 to 2.73]	.96
FGR	4.12 (1.79) [0.60 to 7.63]	.02
Externalizing		
PE	0.69 (1.25) [−1.76 to 3.15]	.58
FGR	2.14 (1.83) [−1.46 to 5.73]	.24
Total		
PE	0.21 (1.37) [−2.48 to 2.91]	.88
FGR	3.06 (1.85) [−0.57 to 6.68]	.10

Abbreviations: FGR, fetal growth restriction; PE, preeclampsia.

^a^ Adjusted models nested infants within multiple-birth families and site. Adjusted models controlled for infant biologic sex, gestational age, hypoaroused and hyperaroused neurobehavior on the NICU Network Neurobehavioral Scale, cumulative medical morbidities on the Bassler index,^28^ partner status, low socioeconomic status based on maternal education and occupation, chorioamnionitis, antenatal steroids, maternal prepregnancy obesity, and maternal age at birth.

^b^ Significant at Bonferroni adjusted α = .008.

The sensitivity analysis, examining whether inclusion of medical morbidities and/or neonatal neurobehavior would influence the results, was performed. Rerunning the adjusted models without these covariates did not substantively change our findings. Thus, medical morbidities and dysregulated neonatal neurobehavior were retained as covariates in the final models (eTable 4 in Supplement 1).

## Discussion

In this cohort study, we examined the associations of preeclampsia and FGR with neurodevelopmental and behavioral outcomes of preterm infants and found no association of preeclampsia with 24-month outcomes, which does not support our primary hypothesis. However, in partial support of our second hypothesis, FGR was significantly associated with adverse developmental outcomes on all 3 Bayley-III scales but was not significantly associated with behavioral outcomes on the CBCL at 24 months corrected age. Our final hypothesis regarding the interaction between preeclampsia and FGR on 24-month outcomes was also unsupported.

Although there were no associations between maternal preeclampsia and 24-month child outcomes in the current study, this work contributes to the literature regarding the impact of preeclampsia on very preterm infant development through concomitantly evaluating FGR and preeclampsia separately and together. Most studies evaluating the association of preeclampsia with child neurodevelopment have been of moderate-to-late preterm (32-36 weeks’ gestation)^38^ and full-term (≥37 weeks’ gestation) infants from large birth or population-based cohorts.^39,40,41,42,43,44,45,46,47,48,49^ Previous research has found that children born at full term to mothers with preeclampsia had a higher risk of neurobehavioral problems and intellectual disability in later childhood,^39^ as well as increased neuropsychiatric outcomes,^13,40,50,51^ increased cognitive impairment,^38,41,42,43^ and increased rates of ADHD diagnoses.^44,45,46,47,48,49,50^ Although 1 study^52^ does report a language delay at 2 years of age in infants born to mothers with a hypertensive disorder of pregnancy, most of these studies are reporting outcomes in later childhood through adulthood in association with maternal preeclampsia. As a result, it remains unclear whether some of these poor outcomes are simply emerging later in childhood and are not able to be detected at an earlier age. A small number of studies have assessed the impact of preeclampsia on preterm infants and reported conflicting findings regarding neurodevelopmental or behavioral outcomes. Children born preterm to mothers with preeclampsia were found to have worse cognitive outcomes at age 2 years compared with preterm infants born to mothers without preeclampsia.^53^ Furthermore, exposure to preeclampsia was found to be an additional negative risk factor in cognitive outcome in preterm infants with intrauterine growth restriction.^54^ Similar to findings in our current study, another cohort of preterm infants found that maternal preeclampsia did not have a significant association with neurodevelopmental outcome.^55^ Our findings do not support an association between maternal preeclampsia and developmental difficulties for preterm infants and adds to the literature attempting to understand the influences of preeclampsia and FGR as early risk exposures and developmental outcomes among very preterm infants.

Infants born to mothers with preeclampsia are deemed to be in a stressful intrauterine environment, which can be further complicated by the experience of FGR.^56^ This suboptimal environment and concomitant effects on the placenta may have a long-lasting impact on the developing fetus,^57,58^ as evidenced by the current findings of FGR being associated with poor long-term developmental outcomes. It is understood that infants born to mothers with preeclampsia are at increased risk of FGR, and in cases of severe preeclampsia, the risk of FGR is even greater.^59^ FGR may be a late marker of preeclampsia severity,^60^ with deleterious effects on delivery of nutrition and oxygen to the fetus. Therefore, the presence of FGR may have differing long-term implications for an infant than an early diagnosis of preeclampsia without FGR. Furthermore, there may be intrauterine environmental differences encountered in FGR with onset earlier in pregnancy leading to preterm delivery, as opposed to late-onset FGR in full-term infants.^61^ Continued research to explore differences between early-onset FGR and late-onset FGR comparing full-term and preterm infants may allow us to better target interventions to improve outcomes for infants who experience FGR and those who are born preterm in relation to intrauterine complications. There is no dispute that there are vast differences in neurodevelopment between preterm and full-term infants, impacted by several antenatal and perinatal covariates,^22,62,63^ and there remains much to understand about how various prenatal insults may or may not manifest themselves later in infant development.

### Strengths and Limitations

Strengths of this study include evaluation of the developmental impact of preeclampsia and FGR on a large multicenter preterm cohort because these infants are already deemed to be at high risk owing to their prematurity. In addition, having data from validated neurodevelopmental assessments by certified examiners, who were not aware of which study participants were exposed to preeclampsia and FGR, allows for unbiased estimates of development in this sample. Importantly, this is one of the first studies to evaluate the effects of preeclampsia and FGR separately and together on early developmental and neurobehavioral outcomes.

One limitation of our study is that we did not have data on certain factors that could impact the association between preeclampsia and neurodevelopmental outcomes, such as timing and number of doses of antenatal corticosteroid administration before delivery. In addition, to ensure standardization of data collection methods among inborn and outborn infants, the diagnosis of preeclampsia was obtained through neonatal medical record reviews of the infant’s prenatal and antepartum history at NICU admission and relied on the accurate reporting of the maternal diagnosis in the neonatal record. Furthermore, the investigators did not have access to Doppler imaging studies, which are commonly used in preeclampsia evaluation and could have provided objective measures of fetal compromise. As such, FGR as defined in our study may include small-for-gestational age infants who may be genetically small without placental insufficiency.^64^ Also, we saw a trend toward higher Bayley-III scores among infants exposed to preeclampsia (eTable 1 in Supplement 1), which may be interpreted as selection bias in that only those infants who were followed-up at 24 months were included in this study. Furthermore, this study sought to evaluate the association of preeclampsia with infant outcomes; however, investigation of other hypertensive disorders of pregnancy or other maternal comorbidities warrant further study.

## Conclusions

In this multicenter preterm infant cohort, there was no association between the presence of preeclampsia and neurodevelopmental outcomes at 24 months corrected age, but FGR was associated with poor neurodevelopmental outcomes. Further investigation is warranted to examine early childhood outcomes associated with the separate and combined effects of FGR with and without preeclampsia and other hypertensive disorders.

## Notes

Supplement 1. **eTable 1.** Unadjusted model examining developmental outcomes at twenty-four months corrected age in infants born to mothers with and without PE **eTable 2.** Unadjusted models examining associations between PE and FGR with 24-month corrected age developmental outcomes **eTable 3.** Adjusted models examining associations between PE, FGR, and PE*FGR interaction with 24-month corrected age developmental outcomes **eTable 4.** Sensitivity analysis examining associations between PE and FGR with 24-month corrected age outcomes without NNNS or medical morbidities

Supplement 2.
Data Sharing Statement
