# Supplementary material for: Preeclampsia, Fetal Growth Restriction, and 24-Month Neurodevelopment in Very Preterm Infants
Source: JAMA Netw Open. 2024 Jul 5;7(7):e2420382. doi: 10.1001/jamanetworkopen.2024.20382 (PMC11227083; doi:10.1001/jamanetworkopen.2024.20382)
Supplement: Supplement 1. — eTable 1. Unadjusted model examining developmental outcomes at twenty-four months corrected age in infants born to mothers with and without PE eTable 2. Unadjusted models examining associations between PE and FGR with 24-month corrected age developmental outcomes eTable 3. Adjusted models examining associations between PE, FGR, and PE*FGR interaction with 24-month corrected age developmental outcomes eTable 4. Sensitivity analysis examining associations between PE and FGR with 24-month corrected age outcomes without NNNS or medical morbidities [file jamanetwopen-e2420382-s001.pdf]

Supplemental Online Content

Check J, Shuster C, Hofheimer J, et al. Preeclampsia, fetal growth restriction, and 24-month neurodevelopment in very preterm infants. *JAMA Netw Open*. 2024;7(7):e2420382. doi:10.1001/jamanetworkopen.2024.20382

eTable 1. Unadjusted model examining developmental outcomes at twenty-four months corrected age in infants born to mothers with and without PE

eTable 2. Unadjusted models examining associations between PE and FGR with 24-month corrected age developmental outcomes

eTable 3. Adjusted models examining associations between PE, FGR, and PE*FGR interaction with 24-month corrected age developmental outcomes

eTable 4. Sensitivity analysis examining associations between PE and FGR with 24-month corrected age outcomes without NNNS or medical morbidities

This supplemental material has been provided by the authors to give readers additional information about their work.

© 2024 Check J et al. *JAMA Network Open.*

eTable 1. Unadjusted model examining developmental outcomes at twenty-four months corrected age in infants born to mothers with and without PE.

	Pre-Eclampsia	n	Estimated Marginal Mean (*SE*)	*B (SE) [95% CI]*	*p*
Bayley-III					
Cognitive Composite	Yes	105	94.57 (1.53)	3.08 (1.73) [-0.32, 6.47]	.08
	No	421	91.50 (0.81)		
Motor Composite	Yes	105	94.08 (1.61)	2.37 (1.77) [-1.09, 5.84]	.18
	No	421	91.70 (0.74)		
Language Composite	Yes	105	90.92 (1.56)	3.44 (1.78) [-0.60, 6.93]	.05
	No	421	87.49 (0.86)		
CBCL					
Internalizing Problems	Yes	103	47.75 (1.11)	-0.47 (1.27) [-2.95, 2.01]	.71
	No	413	47.91 (0.61)		
Externalizing Problems	Yes	103	48.91 (1.05)	-0.51 (1.22) [-2.91, 1.89]	.68
	No	413	49.42 (0.63)		
Total Problems	Yes	103	48.78 (1.15)	-0.80 (1.31) [-3.37, 1.76]	.54
	No	413	49.58 (0.64)		

Note. Models nested infants within sites and multiple-birth families.

© 2024 Check J et al. *JAMA Network Open.*

eTable 2. Unadjusted models examining associations between PE and FGR with 24-month corrected age developmental outcomes.

Unadjusted Model						
	Bayley-III Cognitive		Bayley-III Language		Bayley-III Motor	
	B (SE) [95% CI]	*p*	*B (SE) [95% CI]*	*p*	*B (SE) [95% CI]*	*p*
PE	4.58 (1.72) [1.21, 7.95]	.008[a]	4.75 (1.79) [1.25, 8.25]	.008[a]	3.82 (1.77) [0.34, 7.30]	.031
FGR	-10.62 (2.43) [-15.37, -5.86]	< .001[a]	-9.42 (2.38) [-14.09, -4.76]	< .001[a]	-10.44 (2.49) [-15.31, -5.56]	< .001[a]
	CBCL Internalizing		CBCL Externalizing		CBCL Total	
	B (SE) [95% CI]	*p*	*B (SE) [95% CI]*	*p*	*B (SE) [95% CI]*	*p*
PE	-0.88 (1.31) [-3.46, 1.69]	.50	-0.72 (1.25) [-3.16, 1.72]	.56	-1.12 (1.36) [-3.79, 1.56]	.41
FGR	3.04 (1.75) [-0.39, 6.48]	.08	1.48 (1.86) [-2.18, 5.13]	.43	2.17 (1.88) [-1.52, 5.86]	.25

Abbreviation: PE, preeclampsia; FGR, fetal growth restriction.
Note. Unadjusted model nested infants within multiple-birth families and site.
[a] significant at Bonferroni adjusted α = .008.

© 2024 Check J et al. *JAMA Network Open.*

eTable 3. Adjusted models examining associations between PE, FGR, and PE*FGR interaction with 24-month corrected age developmental outcomes.

Adjusted Model						
	Bayley-III Cognitive		Bayley-III Language		Bayley-III Motor	
	B (SE) [95% CI]	*p*	*B (SE) [95% CI]*	*p*	*B (SE) [95% CI]*	*p*
PE	3.43 (1.65) [-0.19, 6.66]	.04	3.92 (1.77) [0.44, 7.39]	.03	1.86 (1.84) [-1.74, 5.47]	.31
FGR	-8.61 (2.441) [-13.33, -3.89]	< .001[a]	-8.29 (2.38) [-12.95, -3.63]	< .001[a]	-7.60 (2.52) [-12.40, -2.66]	.003[a]
PE *FGR	3.46 (4.56) [-5.48, 12.41]	.45	0.25 (4.53) [-8.63, 9.12]	.96	6.85 (4.99) [-2.93, 16.62]	.17
	CBCL Internalizing		CBCL Externalizing		CBCL Total	
	B (SE) [95% CI]	*p*	*B (SE) [95% CI]*	*p*	*B (SE) [95% CI]*	*p*
PE	-0.08 (1.36) [-2.58, 2.73]	.96	0.69 (1.25) [-1.76, 3.15]	.58	0.21 (1.37) [-2.48, 2.91]	.88
FGR	4.12 (1.79) [0.60, 7.63]	.02	2.14 (1.83) [-1.46, 5.73]	.24	3.06 (1.85) [-0.57, 6.68]	.10
PE *FGR	1.20 (3.42) [-5.49, 7.90]	.73	3.97 (3.61) [-3.11. 11.05]	.27	2.91 (3.59) [-4.12, 9.94]	.42

Abbreviation: PE, preeclampsia; FGR, fetal growth restriction.

Note. Adjusted models nested infants within multiple-birth families and site.

Adjusted models controlled for infant biologic sex, gestational age, hypo- and hyper-aroused neurobehavior on the NNNS, cumulative medical morbidities on the Bassler[28] index, partner status, low socioeconomic status based on maternal education and occupation, chorioamnionitis, antenatal steroids, maternal pre-pregnancy obesity, and maternal age at birth.

[a] significant at Bonferroni adjusted α = .008.

© 2024 Check J et al. *JAMA Network Open.*

eTable 4. Sensitivity analysis examining associations between PE and FGR with 24-month corrected age outcomes without NNNS or medical morbidities.

Adjusted Model						
	Bayley-III Cognitive		Bayley-III Language		Bayley-III Motor	
	B (SE [95% CI]	*p*	*B (SE) [95% CI]*	*P*	*B (SE) [95% CI]*	*p*
PE	3.10 (1.64) [-0.11, 6.31]	.06	3.40 (1.75) [-0.03, 6.83]	.05	1.66 (1.80) [-1.87, 5.20]	.36
FGR	-9.85 (2.36) [-14.47, -5.23]	< .001[a]	-9.02 (2.30 [-13.53, -4.50]	< .001[a]	-9.71 (2.48) [-14.58, -4.85]	< .001[a]
	CBCL Internalizing		CBCL Externalizing		CBCL Total	
	B (SE) [95% CI]	*p*	*B (SE) [95% CI]*	*p*	*B (SE) [95% CI]*	*p*
PE	0.09 (1.35) [-2.55, 2.73]	.95	0.38 (1.27) [-2.11, 2.86]	.77	0.04 (1.37) [-2.64, 2.72]	.98
FGR	3.74 (1.77) [0.27, 7.21]	.04	1.72 (1.81) [-1.83, 5.26]	.34	2.57 (1.83) [-1.02, 6.17]	.16

Abbreviation: PE, preeclampsia; FGR, fetal growth restriction; NNNS, NICU Network Neurobehavioral Scale.

Note. Adjusted models nested infants within multiple-birth families and site, while controlling for infant biological sex, gestational age, partner status, low socioeconomic status based on maternal education and occupation, chorioamnionitis, antenatal steroids, maternal pre-pregnancy obesity, and maternal age at birth.

[a] significant at Bonferroni adjusted $\alpha = .008$.

© 2024 Check J et al. *JAMA Network Open.*
