# Supplementary material for: Preeclampsia, Fetal Growth Restriction, and 24-Month Neurodevelopment in Very Preterm Infants
Source: JAMA Netw Open. 2024 Jul 5;7(7):e2420382. doi: 10.1001/jamanetworkopen.2024.20382 (PMC11227083; doi:10.1001/jamanetworkopen.2024.20382)
Supplement: Supplement 2. — Data Sharing Statement [file jamanetwopen-e2420382-s002.pdf]

Data Sharing Statement

Check. Preeclampsia, Fetal Growth Restriction, and 24-Month Neurodevelopment in Very Preterm Infants. *JAMA Netw Open*. Published July 05, 2024.
doi:10.1001/jamanetworkopen.2024.20382

Data
Data available: No

Additional Information
Explanation for why data not available: The datasets generated during and/or analyzed during the current study are not publicly available, but are available from the corresponding author on reasonable request.
